# Analyses of the effects of Gq protein on the activated states of the muscarinic M_3_ receptor and the purinergic P2Y_1_ receptor

**DOI:** 10.1002/phy2.134

**Published:** 2013-10-27

**Authors:** Michihiro Tateyama, Yoshihiro Kubo

**Affiliations:** 1 Division of Biophysics and Neurobiology, Department of Molecular Physiology, National Institute for Physiological SciencesMyodaiji, Okazaki, 444-8585, Aichi, Japan; 2 Department of Physiological Sciences, School of Life Science, The Graduate University for Advanced Studies (SOKENDAI)Myodaiji, Okazaki, 444-8585, Aichi, Japan

**Keywords:** FRET, G protein, GPCR

## Abstract

G protein–coupled receptors (GPCRs) cause various cellular responses through activating heterotrimeric G protein upon the agonist binding. The interaction with G protein has been suggested to stabilize the agonist-bound active conformation of GPCRs. We previously reported the effects of Gq protein on the stabilization of the active conformation of the muscarinic receptor type 1 (M_1_R), using a fluorescence resonance energy transfer (FRET) technique. In this study, we aimed at examining whether or not the binding of Gq protein affects the agonist-induced active conformation of receptors other than the M_1_R. For this purpose, functionally intact fluorescent receptors of the metabotropic purinergic receptor type 1 (P2Y_1_R) and muscarinic receptor type 3 (M_3_R) were constructed, by inserting junctional linkers between the short intracellular third loops (i3) and yellow fluorescent protein (YFP). The YFP-fused receptors also showed the agonist-induced increases in FRET from the cyan fluorescent protein (CFP) tethered with G_αq_ subunit, indicating that they interacted with Gq protein. The agonist-induced conformational changes of the receptors were detected as the agonist-induced decrease in FRET between YFP at the i3 and CFP at the C-tail. The FRET decrease of the M_3_R but not of the P2Y_1_R was enhanced by coexpression of Gq protein. In addition, coexpression of Gq protein significantly decelerated the FRET recovery of the M_3_R construct but not of the P2Y_1_R construct upon the agonist removal. These results suggest that the effects of the Gq binding on the active conformation of the receptor differ depending on the type of GPCRs.

## Introduction

Neurotransmitters, neuropeptides, and hormones modulate the cellular activity and function through selective binding to G protein-coupled receptors (GPCRs). Binding of those biological molecules induces conformational changes of GPCRs, resulting in the coupling with heterotrimeric G protein. The interaction leads to dissociation of the *α* subunit and the accessory βγ subunits of G protein and to initiate the downstream signaling (Tesmer 2010). Recent studies of the X-ray crystallography solved the ligand-bound structures of GPCR, such as the antagonist- or agonist-bound structures of receptors (Cherezov et al. 2007; Haga et al. 2012; Kruse et al. 2012; Lebon et al. 2011; Rasmussen et al. 2011a; Rosenbaum et al. 2007) and the structure of the agonist/β-adrenoceptor/Gs complex (Rasmussen et al. 2011b). The structural analyses conferred insights into the mechanism how the ligand-binding at the transmembrane (TM) domain of GPCR induces the interaction and the activation of G protein.

The transition between the different conformational states of GPCR can be analyzed by using the optical methods. The agonist-induced conformational changes in GPCR have been monitored as the agonist-induced changes in efficiency of fluorescence resonance energy transfer (FRET) between the fluorescent proteins (FPs) tethered at the intracellular domains of the receptors (Hoffmann et al. 2005; Markovic et al. 2012; Matsushita et al. 2010; Tateyama et al. 2004; Vilardaga et al. 2003). Because the FPs are tethered at the intracellular domains, the agonist-induced FRET changes are thought to reflect the agonist-induced rearrangements of the intracellular domains which allow G protein coupling. Binding of G protein to the receptors has been suggested to stabilize the agonist-induced active conformation of GPCR (Christopoulos and Kenakin 2002), with evidences that the inhibition of G protein coupling attenuates the agonist binding. FRET studies also reported that binding of G protein affects the agonist-induced active conformations (Tateyama and Kubo 2013; Vilardaga et al. 2003). In our previous FRET study of the muscarinic receptor type 1 (M_1_R), Gq protein enhanced the agonist-induced decrease in FRET between FPs at the third intracellular loop (i3) and the C-tail. In addition, the binding of Gq protein decelerated the recovery of the decreased FRET upon the agonist washout. These results were consistent with the notion that the active conformation of the receptor is stabilized by the G protein binding (Rasmussen et al. 2011a,b). However, the effects of the Gq binding on the agonist-induced active conformation were not examined in Gq-coupled receptors other than the M_1_R.

Here, we investigated the effects of the Gq binding on the active conformation of the metabotropic purinergic receptor type 1 (P2Y_1_R) and muscarinic receptor type 3 (M_3_R). By inserting yellow fluorescent protein (YFP) into the short i3 with junctional linkers, we obtained fluorescent constructs which can functionally interact with Gq protein. FRET studies revealed that Gq protein significantly enhanced the agonist-induced FRET decrease of the M_3_R construct but not of the P2Y_1_R construct. These results suggested that the stabilizing effects of Gq protein on the active conformation of the receptors differ depending on the type of the coupled receptors.

## Material and Methods

### Constructs and expression system

**Figure 1:**
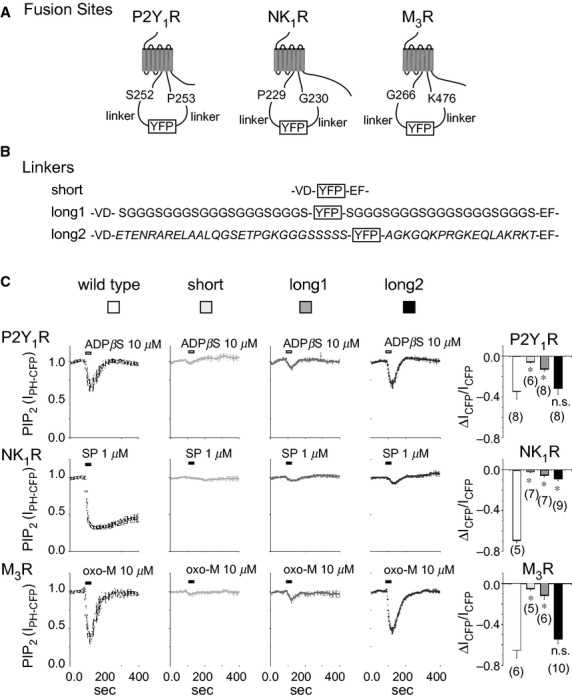
The long linkers between the i3 and YFP restored the functional Gq coupling of the P2Y_1_R, M_3_R but not of NK_1_R. (A) Shown are the YFP fusion sites of the P2Y_1_R, NK_1_R and M_3_R. (B) Sequences of the junctional linkers are shown. The long1 linker is flexible GGGS repeat and the long2 linker is derived partly from the i3 of the M_1_R (shown in italic). (C) Gq coupling of the i3-YFP receptors. Functional Gq coupling of the constructs was evaluated as decreases in the emission intensity of CFP tethered to the PH domain (*I*_CFP-PH_) under TIRF illumination. Traces represent the time laps changes in the *I*_CFP_ normalized by the baseline *I*_CFP_. Black bar over each trace represents the application of the ADPβS (10 μmol/L) for P2Y_1_R, substance P (SP, 1 μmol/L) for the NK_1_R or oxo-M (10 μmol/L) for the M_3_R. Bars in the right panels represent the mean of the maximal decreases in the normalized *I*_CFP_. Numbers of experiments are shown in parentheses. The long2 linker restored the Gq coupling of the P2Y_1_R-i3-YFP and the M_3_R-i3-YFP, but not of the NK_1_R-i3-YFP. *P < 0.05, n.s., not significant; i3, intracellular third loops; NK_1_R, neurokinin receptor type 1; P2Y_1_R, the metabotropic purinergic receptor type 1; M_3_R, muscarinic receptor type 3.

Complementary DNAs for mouse G_αq_, G_β1_, and G_γ2_ subunits and mouse P2Y_1_R were isolated as previously described (Tateyama and Kubo 2013). The G_aq_-CFP was kindly gifted from Dr. Berlot (Witherow et al. 2003) and the cyan fluorescent protein (CFP) tethered with PH domain (CFP-PH) was from Dr. Jalink (van der Wal et al. 2001). Restriction enzyme sites (*Sal*I and *Eco*RI) with or without junctional linker sequences were introduced before and after the coding regions (Val2-Lys239) of YFP, respectively, by polymerase chain reaction (PCR) with designed primers and KOD plus Neo polymerase (Toyobo, Osaka, Japan). Those restriction enzyme sites were also introduced at the i3 of the human and the mouse P2Y_1_Rs, the rat M_3_R and the mouse neurokinin receptor type 1 (NK_1_R) (see Fig. 1A). Then the amplified fragments including YFP coding regions were inserted into the i3 of the receptors. CFP was then fused at the C-tail of the i3-YFP constructs, as previously described (Tateyama and Kubo 2013). After confirmation of the sequence (ABI 3130x, Carlsbad, CA), each construct was subcloned into the pcDNA3.1(−) expression vector. Human embryonic kidney 293T (HEK293T) cells were transfected with the plasmid DNA and the transfected cells were seeded onto glass bottom dishes, as previously reported (Tateyama and Kubo 2013). Experiments were carried out 24–48 h after transfection. Before imaging, cells were incubated for more than 30 min in Hank's balanced salt solution (Invitrogen, Carlsbad, CA) supplemented with 1 mmol/L Ca^2+^ and 0.3 mmol/L Mg^2+^ at room temperature.

### Imaging

Fluorescence from single cell expressing the fluorescent constructs was imaged and measured using a total internal reflection fluorescence (TIRF) microscope (Olympus, Tokyo, Japan). Cells were continuously perfused with bath solution (140 mmol/L NaCl, 1 mmol/L CaCl_2_, 4 mmol/L KCl, 0.3 mmol/L MgCl_2_, 10 mmol/L HEPES, and pH 7.4 adjusted with NaOH) by gravity at a rate of about 3 mL/min, and each concentration of agonists was applied by changing the perfusion solution. CFP and YFP were excited by the 442 and 515 nm laser lines, respectively. The emitted light was passed through S470/30 and S535/30 filters (Chroma, Bellows Falls, VT), respectively. The fluorescence images were then amplified by an image intensifier unit (C8600, Hamamatsu Photonics, Hamamatsu, Japan) and recorded every 3 sec using a cooled CCD camera (Micromax, Roper Scientific, Tucson, AZ). A laser switching controller and the image acquisition were operated by using MetaFluor imaging software (Molecular Devices, Sunnyvale, CA). The exposure time was 200 msec for CFP&FRET and 50 msec for YFP. Emission intensities of FRET (*I*_FRET_), CFP (*I*_CFP_), and YFP (*I*_YFP_) were measured by subtracting their background intensities. To calculate the FRET efficiency, intensities of the bleed-through fraction of CFP and YFP were subtracted from the intensity of FRET (net FRET, nF = *I*_FRET_ − 0.40 × *I*_CFP_ − 0.058 × *I*_YFP_) and then the nF was normalized by the intensity of CFP (nF/*I*_CFP_).

### Fluorescence photometry

For high time resolution analyses, we carried out the photometry. Cells seeded on the glass bottom dish were placed on inverted microscope and continuously epi-illuminated by xenon lamp. CFP of the constructs was excited by light pass through a band-path excitation filter (426–450 nm). Emitted light was divided by a 505 mirror and the intensity of shorter light pass through an emission filter (430–490 nm) was detected by a photomultiplier tube. That of longer light pass through an emission filter (510–550 nm) was detected by a different photomultiplier tube. The former corresponds to *I*_CFP_ and the latter to *I*_FRET_. The emission intensities were simultaneously acquired as voltage by the digidata and pClamp 9 software (Axon Instruments, Foster City, CA) with a sampling frequency of 2 kHz. Solution exchange was performed by lateral movement of a theta glass equipped with a step-driven motor. Time for the solution exchange was evaluated by monitoring the changes in junction potential: 140 mmol/L Na^+^ in a bath solution was exchanged to 140 mmol/L K^+^ within 20 msec. Baseline of *I*_FRET_ was adjusted to cancel the quenching-induced decline and then the ratio of *I*_FRET_ to *I*_CFP_ was calculated (FRET ratio, *I*_FRET_/*I*_CFP_). Changes in the FRET ratio upon the application and the removal of the agonist were fitted to a single exponential function to elucidate the kinetics of the conformational changes.

### Analysis and statistics

The baseline *I*_CFP-PH_ and baseline nF/*I*_CFP_ were the averages of *I*_CFP-PH_ and nF/*I*_CFP_ for 13 time points before the agonist application, respectively. To evaluate the intact function of wild type and the fluorescent receptors, the maximal decrease in the *I*_CFP-PH_ after the agonist application was normalized to the baseline value of the *I*_CFP-PH_ in each cell. As for the ligand-induced decrease in FRET (ΔnF/*I*_CFP_), values of nF/*I*_CFP_ from the third to the tenth time points after ligand application were averaged in each experiment. Various concentrations of agonists were applied to cells via perfusion to evaluate the relationship between concentration and response. The amplitudes of the ΔnF/*I*_CFP_ were normalized to those obtained by the highest concentration of the agonists in each cell. The EC_50_ values were estimated by fitting the concentration–response plot to a Hill equation (Origin8; OriginLab, Northampton, MA). All data are expressed as means ± SE, with n indicating the number of data. A statistical significance between two groups was estimated by unpaired Student's *t*-test and that between more than two groups was done by a one-way analysis of variance (ANOVA) followed by Dunnett's *t*-test; values of *P* < 0.05 were considered statistically significant.

## Results

### The functional Gq coupling of the i3-YFP constructs

In this study, we aimed at investigating the effects of the Gq binding on the active conformation of the receptors. For this purpose, it is required that fusion of FP to the intracellular loops of the receptors does not disrupt the interaction with Gq protein although it abolished the interaction in some cases reported previously (Tateyama et al. 2004; Jensen et al. 2009; Matsushita et al. 2010). From recent FRET studies of the M_1_R, distance from the FP to the cytoplasmic ends of TM5 and TM6 was suggested to be critical for the Gq coupling. The fluorescent M_1_R constructs were not functional when numbers of the amino acid (a.a.) residues linking the FP with the TM5 or TM6 were less than 20 (Jensen et al. 2009), whereas they were functional when the numbers were more than 30 (Markovic et al. 2012; Tateyama and Kubo 2013). These results suggested that the long linkers serve as spacers that allow the receptors to couple with Gq protein. YFP was then inserted into the intrinsically short i3 of the P2Y_1_R and NK_1_R with or without junctional linkers. As the junctional linker, the flexible GlyGlyGlySer (GGGS) repeats or parts of amino acid sequence of the i3 of the M_1_R were selected (Fig. 1B). We also replaced most of the long i3 of the M_3_R with YFP and inserted the junctional linkers, to examine whether or not the linker mimic the endogenous i3 of the M_3_R (Fig. 1 A and B). The expression on the surface membrane was examined by measuring the *I*_YFP_ under the TIRF illumination (Tateyama and Kubo 2011) and was confirmed in all i3-YFP receptors (data not shown).

The intact Gq coupling of the i3-YFP receptors was examined by monitoring changes in the emission intensity of CFP tethered with the PH domain (*I*_CFP-PH_) under the TIRF illumination. As the PH domain interacts with PIP_2_, PIP_2_ breakdown induced by the activation of Gq signaling pathway disrupts the membrane localization of the CFP-PH (van der Wal et al. 2001). In fact, application of agonists, such as the ADPβS (10 μmol/L), Substance P (SP, 1 μmol/L), or oxo-M (10 μmol/L), induced decreases in the *I*_CFP-PH_ under the TIRF illumination in cells transfected with wild-type P2Y_1_R, NK_1_R, or M_3_R, respectively (Fig. 1C traces in left panels). Although the P2YRs are known to be endogenously expressed in the HEK293T cells, their contribution to the decreases in the *I*_CFP-PH_ could be ignored (data not shown).

When YFP was inserted into the short i3 of the P2Y_1_R and NK_1_R, application of the agonists failed to decrease the *I*_CFP-PH_. Similarly, activation of the M_3_R-i3-YFP failed to trigger the Gq pathway when most of the i3 was replaced with YFP (M_3_R-i3-YFP-short). In contrast, decreases in the *I*_CFP-PH_ were observed upon the agonist application in the i3-YFP receptors with the junctional linkers. These results supported that the long linkers serve as spacers. However, the maximal amplitudes of decreases in the *I*_CFP-PH_ (Δ*I*_CFP-PH_) were different between the i3-YFP-long1 and -long2 constructs. As the junctional linkers derived from M_1_R restored the intact function comparable to that of wild-type receptors, the i3-YFP-long2 constructs of P2Y_1_R and M_3_R were used for further analyses. In the case of the NK_1_R, the construct with long2 junctional linker only partially and transiently triggered the Gq pathway. The change was not comparable to the phenotype of wild-type receptors. Thus, the NK_1_R constructs were not used for further studies.

Between the P2Y_1_R-i3-YFP-long2 and the M_3_R-i3-YFP-long2, the maximal amplitudes of the Δ*I*_CFP-PH_ were different. The Δ*I*_CFP-PH_ induced by the P2Y_1_R-i3-YFP-long2 was almost half of that by the M_3_R-i3-YFP-long2. The basal intensities of the *I*_CFP_ in cells expressing the i3-YFP-long2 constructs were not different and the surface expression of the P2Y_1_R-i3-YFP-long2 was not smaller than that of the M_3_R- i3-YFP-long2 (data not shown). Therefore, the difference in the maximal amplitudes of Δ*I*_CFP-PH_ was thought to possibly reflect the difference in efficiency of the Gq coupling of the i3-YFP-long receptors.

### FRET analyses between the i3-YFP constructs and the G_αq_-CFP subunits

**Figure 2:**
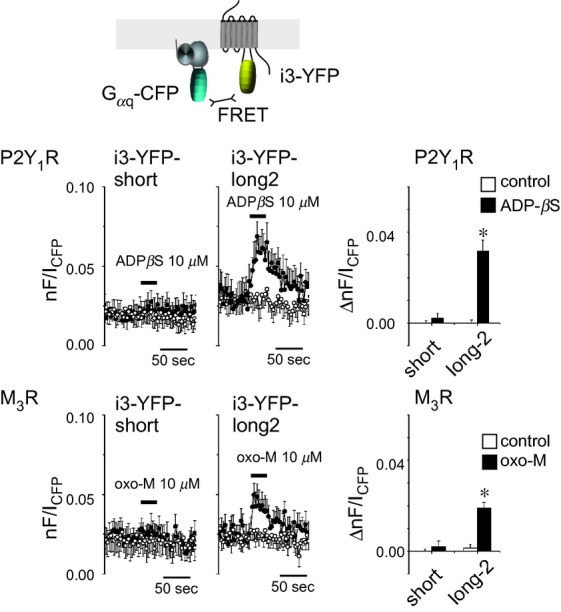
FRET analyses of the interaction between the receptor-i3-YFP and G_*α*q_-CFP. A schematic drawing of the monitored FRET is shown in the top panel. Traces represent the time-lapse changes in FRET between the receptor-i3-YFP and the G_*α*q_-CFP in the presence of G_β1_ and G_γ2_ (left panels). The efficiency of FRET at each time point was calculated as the nF/*I*_CFP_ in each experiment (see Material and Methods) and then averaged (*n* = 9–13). Agonists or vehicle were applied for 30 sec to cells expressing the fluorescent constructs (filled bars on traces). The change in FRET (ΔnF/*I*_CFP_) was calculated by subtracting the basal nF/*I*_CFP_ value before the application from the nF/*I*_CFP_ after the application in each experiment. Averaged ΔnF/*I*_CFP_ is shown as bar in the right panels (*n* = 9–13). FRET, fluorescence resonance energy transfer. *P < 0.05.

The interaction of the constructed receptors with Gq protein was also examined by monitoring FRET between the i3-YFP receptors and the G_*α*q_-CFP, as we previously reported (Tateyama and Kubo 2013). When the i3-YFP-short constructs were expressed with the G_*α*q_-CFP and G_β1_G_γ2_ subunits, FRET were not changed by the application of the agonists (Fig. 2). In contrast, the nF/*I*_CFP_ were significantly increased upon the application of the agonists when the i3-YFP-long2 constructs of the P2Y_1_R and the M_3_R were coexpressed with the G_*α*q_-CFP and G_β1_Gγ_2_ subunits (Fig. 2). These results demonstrated that the i3-YFP-long2 constructs but not clearly the short ones interacted with Gq protein upon the application of the agonist, which goes well with the results obtained from the functional experiments (Fig. 1).

### The agonist-induced decreases in the intrasubunit FRET and the effects of Gq protein on the decreases in FRET

**Figure 3:**
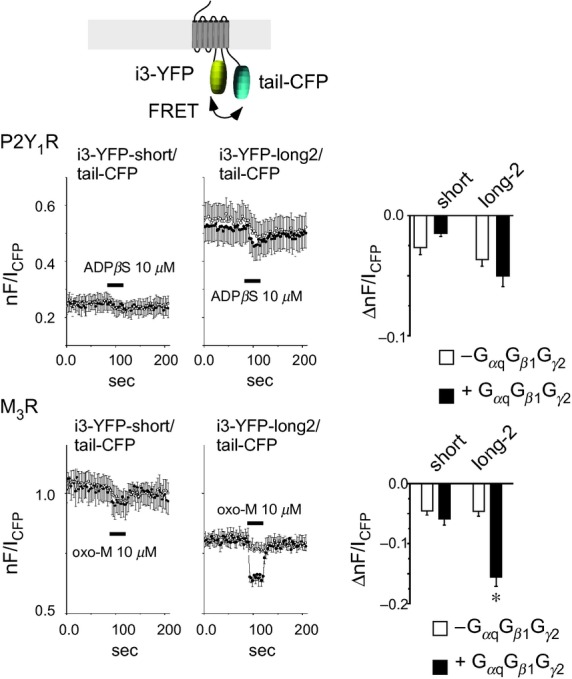
Coexpression of the G_*α*q_G_β1_G_γ2_ subunits differentially affected the agonist-induced FRET decreases of the P2Y_1_R and M_3_R FRET constructs. A schematic drawing of the monitored FRET is shown in the top panel. Traces in the left panels represent the time-lapse changes in FRET of the indicated constructs with (filled symbols) or without (open symbols) coexpression of Gq protein. Agonists were applied for 30 sec (black bars on traces) to cells expressing the fluorescent receptors. The changes in FRET (ΔnF/*I*_CFP_) were calculated by subtracting the basal nF/*I*_CFP_ values before application from those after application (see Material and Methods). Averaged ΔnF/*I*_CFP_ is shown as bar in the right panels (*n* = 8–16). FRET, fluorescence resonance energy transfer; P2Y_1_R, the metabotropic purinergic receptor type 1; M_3_R, muscarinic receptor type 3; ΔnF/*I*_CFP_, ligand-induced FRET changes. *P < 0.05.

**Figure 4:**
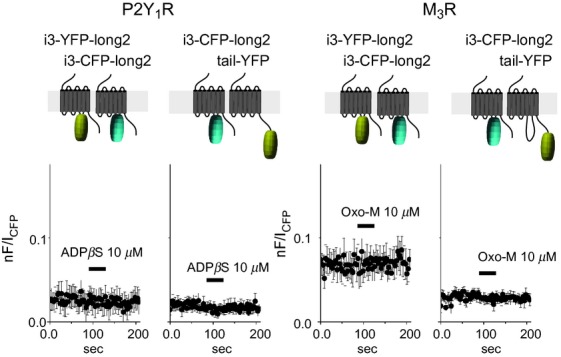
FRET between the fluorescent P2Y_1_R subunits and between the fluorescent M_3_R subunits was not changed upon the agonist application. FRET between the FP fused receptors under the TIRF illumination were measured. Combination of the expressed constructs is shown in the upper panels and the time-lapse changes in FRET are shown. The application of the agonists (shown in black bars upper traces) did not alter the efficiency of the FRET between the i3-YFP and i3-CFP and that between the i3-YFP and tail-CFP (*n* = 6–8). FRET, fluorescence resonance energy transfer; P2Y_1_R, the metabotropic purinergic receptor type 1; M_3_R, muscarinic receptor type 3; FP, fluorescent protein; TIRF, total internal reflection fluorescence; i3, intracellular third loop.

As a next step, CFP was attached at the C-terminus of the i3-YFP constructs to monitor their conformational changes as the FRET changes. Upon application of the agonists, decreases in FRET were detected in all tested constructs (Fig. 3). The agonist-induced decreases in FRET were not observed in the presence of the antagonists (Table 1), indicating that the changes in FRET were induced by the binding of the agonists. The agonist-induced decreases in FRET were thought to reflect not only the conformational changes of the receptor but also possibly the rearrangements of the receptor oligomers, as the P2Y_1_R and the M_3_R have been reported to form oligomers (Zeng and Wess 1999; Choi et al. 2008). However, FRET between the fluorescent receptors was not changed by the agonist application either in the P2Y_1_R or the M_3_R (Fig. 4), indicating that the agonist-induced FRET decreases observed in the P2Y_1_R-i3-YFP/tail-CFPs and the M_3_R i3-YFP/tail-CFPs reflect mostly the conformational change of the protomer but not of the rearrangements between the protomers. The decreases in the FRET efficiency were consistent with the outward movements of YFP at the i3 from CFP at the C-tail as shown in β-adrenergic receptor (Rasmussen et al. 2011b).

**Table 1:** Antagonists inhibited the agonist-induced FRET decreases.

	P2Y_1_R-i3-YFP-long1/tail-CFP	P2Y_1_R-i3-YFP-long2/tail-CFP
ΔnF/*I*_CFP_ by ADPβS 10 μmol/L	0.030 ± 0.004 (3)	0.031 ± 0.004 (7)
With MRS2179 20 μmol/L	0.008 ± 0.004 (4)*	0.005 ± 0.005 (5)*

	M_3_R-i3-YFP-long1/tail-CFP	M_3_R-i3-YFP-long2/tail-CFP
ΔnF/*I*_CFP_ by oxo-M 10 μmol/L	0.041 ± 0.010 (3)	0.032 ± 0.004 (4)
With atropine 50 μmol/L	0.006 ± 0.008 (4)*	0.005 ± 0.005 (4)*

Values of ΔnF/*I*_CFP_ were evaluated at 10 μmol/L ADPβS for the P2Y_1_R construct and at 10 μmol/L oxo-M for the M_3_R construct in the presence or absence of the antagonists. The antagonists, 20 μmol/L MRS2179 for the P2Y_1_R constructs and 50 μmol/L atropine for the M3R constructs, were preapplied 30 sec before the agonist application and also coapplied with the agonists. Mean and SE values are shown. Numbers of experiments are indicated in parentheses.

* *P* < 0.05.

**Figure 5:**
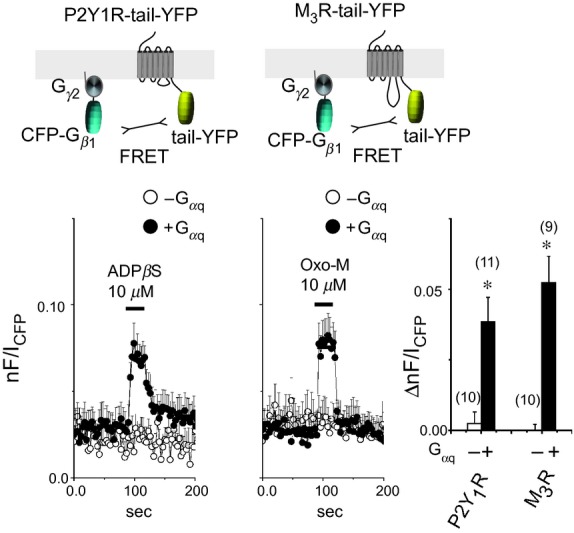
The interaction between the receptor-YFP and G_β1_ subunits in the presence or absence of the exogenously expressed G_*α*q_. FRET analyses of the interaction between the receptor-tail-YFP and CFP-G_β1_ in the presence or absence of the exogenously expressed G_*α*q_ subunit. In the experiments, the Gγ_2_ subunit was also cotransfected with the CFP-G_β1_. For the FRET analysis, cells expressing sufficient FP fused constructs were selected, to minimize the fluctuation of the small baseline FRET values. Traces represent the time-lapse changes in FRET between the tail-YFP and the CFP- G_β1_. Application of agonists increased the FRET efficiency when the G_*α*q_ subunit was cotransfected, while it failed to increase the FRET efficiency when the G_*α*q_ subunit was not cotransfected. The results indicate that the contents of the endogenous Gq protein were not sufficient to interact with the exogenously expressed fluorescent receptor. Shown are average and SE. Numbers of experiments are shown in parentheses. FRET, fluorescence resonance energy transfer. *P < 0.05 comparison between −G_*α*q_ and +G_*α*q_.

Some of the FRET constructs are expected to form complexes of agonist/Receptor/Gq (A/R*/Gq, R*: indicates activated receptor), as the i3-long2-YFP/tail-CFP receptors are able to interact with Gq protein. However, a major fraction of the i3-long2-YFP/tail-CFP receptors would not form the A/R*/Gq complex when transfected alone, as the amount of endogenous Gq protein was not sufficient to bind to the exogenously over expressed P2Y_1_R and the M_3_R constructs (Fig. 5). Therefore, the agonist-induced FRET decreases were thought to reflect mostly the agonist-bound active conformation of the receptors (A/R*), when the FRET constructs were transfected alone.

The effects of the Gq binding on the active conformation of the receptors were examined by coexpression of the G_*α*q_G_β1_G_γ2_ subunits with the FRET constructs. When Gq protein was coexpressed with i3-YFP-short/tail-CFP constructs, the ΔnF/*I*_CFP_ were not changed in the P2Y_1_R and M_3_R (Fig. 3 right panels). These results corresponded to the previous results that the i3-YFP-short constructs did not interact with Gq protein. On the other hand, coexpression of the G_*α*q_G_β1_G_γ2_ subunits significantly increased the ΔnF/*I*_CFP_ of M_3_R-i3-YFP-long2/tail-CFP but not of P2Y_1_R-i3-YFP-long2/tail-CFP (Fig. 3 center panels and Table2). As for the M_3_R construct, the constitutively active mutant (Q209L) of the G_*α*q_ subunit was coexpressed instead of wild-type one (Qian et al. 1993) to examine the effects of the GTP-bound form of the G_*α*q_ subunit on the active conformation of the M_3_R. The ΔnF/*I*_CFP_ with G_*α*q_-Q209L was 0.068 ± 0.008 (*n* = 6), which was comparable to that without exogeneous Gq protein (0.056 ± 0.01, *n* = 9). Lack of the enhancement of the ΔnF/*I*_CFP_ by the G_*α*q_-Q209L was consistent with the notion that the GTP-bound G_*α*q_ does not bind to the M_3_R.

**Table 2:** Parameters of FRET change analysis of the i3-YFP-long2/tail-CFP constructs with or without coexpression of G_*α*q_G_β1_G+_2_ subunits.

		EC_50_ (μmol/L)	ΔnF/*I*_CFP_
P2Y_1_R constructs (ADPβS)	−Gq	0.36 ± 0.15 (5)	0.032 ± 0.003 (5)
	+Gq	0.28 ± 0.07 (6)	0.037 ± 0.003 (6)
M_3_R constructs (oxo-M)	−Gq	3.14 ± 1.18 (4)	0.052 ± 0.005 (4)
	+Gq	0.89 ± 0.25* (4)	0.133 ± 0.016* (4)

Values of ΔnF/*I*_CFP_ were evaluated at 10 μmol/L ADPβS for the P2Y_1_R construct and at 10 μmol/L oxo-M for the M_3_R construct. Mean and SE values are shown. Numbers of experiments are indicated in parentheses.

* *P* < 0.05.

In the case of the P2Y_1_R construct, coexpression of the Gq subunits did not significantly increase the ΔnF/*I*_CFP_. Similar results were observed (1) when fusion sites of two FPs were exchanged (P2Y_1_R-i3-CFP-long2/tail-YFP, data not shown), (2) when a full agonist (2MeSADP, 10 μmol/L) was applied (Table3), and (3) when the mouse, not human, P2Y_1_R-i3-YFP-long2/tail-CFP was transfected with the mouse Gq subunits (Table4). These results suggested that the lack of the Gq-induced enhancement of the ΔnF/*I*_CFP_ was not due to the difference either in (1) the fusion sites of FPs or in (2) the type of agonists or by (3) the mismatch of the human P2Y_1_R and the mouse Gq subunits. The lack of the Gq-induced enhancement of the ΔnF/*I*_CFP_ might be due to disruption of the coupling to Gq protein by the additional FP fusion at the C-tail. We examined this possibility by monitoring the *I*_CFP-PH_ and measuring the FRET efficiency against the G_*α*q_-CFP in cells expressing the P2Y_1_R-i3-YFP-long2/tail-HT in which CFP of the P2Y_1_R-i3-YFP-long2/tail-CFP was replaced with a bulky construct of halotag (HT). In cells expressing P2Y_1_R-i3-YFP-long2/tail-HT, application of the ADPβS (10 μM) markedly decreased the *I*_CFP-PH_ (Δ*I*_CFP-PH_ was 41 ± 11%, *n* = 4). In addition, the significant increase in FRET between the P2Y_1_R-i3-YFP-long2/tail-HT and the G_*α*q_-CFP was observed; ΔnF/*I*_CFP_ by ADPβS 10 μmo/L was 0.014 ± 0.002 (*n* = 9) and that by vehicle was 0.001 ± 0.003 (*n* = 6, *P* < 0.01). Because the size of HT is similar to that of CFP, it appears that Gq protein bound to the P2Y_1_R-i3-YFP-long2/tail-CFP without significantly increasing the ΔnF/*I*_CFP_ upon the agonist application.

**Table 3:** Parameters of the 2MeSADP-induced changes in FRET for the human P2Y1R-i3-YFP-long2/tail-CFP constructs with or without coexpression of G_*α*q_G_β1_G_γ2_ subunits.

		ΔnF/*I*_CFP_	τ_act_ (mS)	τ_rec_ (mS)
P2Y1R-i3-YFP-long2/tail-CFP (2MeSADP 10 μmol/L)	−Gq	0.026 ± 0.004 (10)	696 ± 108 (5)	2215 ± 276 (5)
	+Gq	0.035 ± 0.006 (8)	501 ± 47 (5)	2986 ± 548 (5)

Values of ΔnF/*I*_CFP_, τ_act_, and τ_rec_ were evaluated at 10 μmol/L 2MeSADP for the human P2Y_1_R construct. These FRET parameters were not significantly changed by the coexpression of the Gq subunits. Mean and SE values are shown. Numbers of experiments are indicated in parentheses.

**Table 4:** Parameters of FRET change analysis of the mouse P2Y_1_R-i3-YFP-long2/tail-CFP constructs with or without coexpression of G_*α*q_G_β1_G_γ2_ subunits.

		−Gq	+Gq
Mouse P2Y_1_R constructs (ADPβS)	ΔnF/*I*_CFP_	0.031 ± 0.005 (9)	0.040 ± 0.005 (9)

Values of ΔnF/*I*_CFP_ were evaluated at 10 μmol/L ADPβS for the mouse P2Y_1_R construct. Mean and SE values are shown. Numbers of experiments are indicated in parentheses.

**Figure 6:**
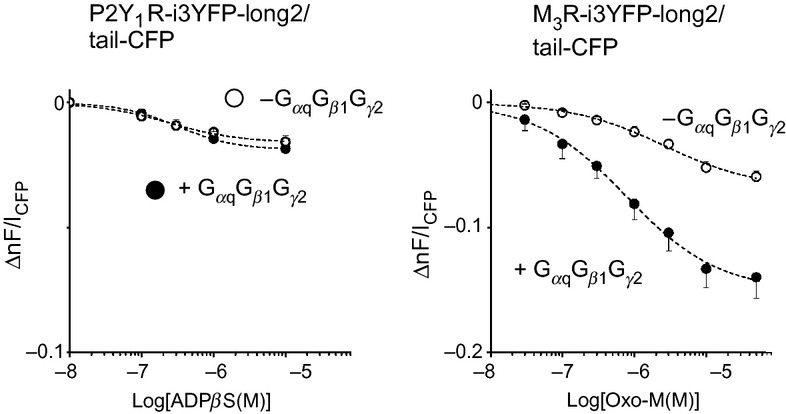
Concentration–response curves of the agonist-induced FRET decreases. Concentration–response curves of the indicated FRET constructs in the presence (filled symbols) or the absence (open symbols) of Gq protein are shown. Values of EC_50_ (μmol/L) and ΔnF/*I*_CFP_ by agonists (10 μmol/L) are summarized in Table2. FRET, fluorescence resonance energy transfer; ΔnF/*I*_CFP_, ligand-induced FRET changes.

The Gq-induced enhancement of the ΔnF/I_CFP_ in the M_3_R was observed over various agonist concentrations (Fig. 6) and the Gq binding significantly shifted the concentration–response curve of the M_3_R-i3-YFP-long2/tail-CFP toward the left (Fig. 6 and Table2). In contrast, coexpression of the Gq subunits did not increase the ΔnF/I_CFP_ in the P2Y_1_R at various concentrations (Fig. 6). From the concentration–response curve, the EC_50_ values were roughly estimated and were not significantly different between the two cases (Table2). These results suggested that the binding of Gq protein exerts different effects on the active conformation of the P2Y_1_R and M_3_R.

### Effects of Gq protein on the kinetics of the transition between the quiescent and active conformation of the receptors

**Figure 7:**
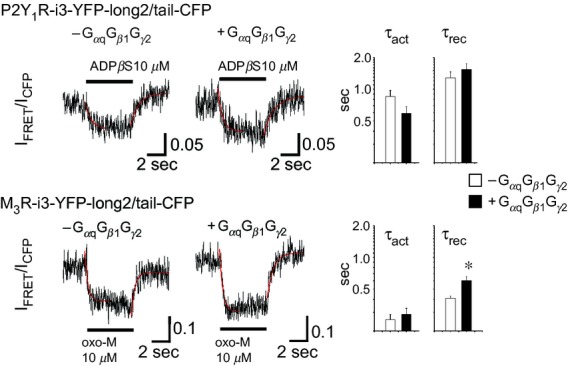
Kinetic analyses of the activation and deactivation of the receptors. The emission intensities of CFP (*I*_CFP_) and FRET (*I*_FRET_) were monitored by high time resolution fluorescence photometry. Time-lapse changes in the calculated FRET (*I*_FRET_/*I*_CFP_) are shown in the left panels. The indicated agonists were applied for 5 sec through the fast perfusion system. The decline and recovery phases of FRET changes were fitted to a single exponential function to estimate the time constants (τ_act_ and τ_rec_, respectively). The values are shown as bars on the right (*n* = 6–9). Statistical significance was evaluated by unpaired *t*-test. FRET, fluorescence resonance energy transfer. *P < 0.05.

Binding of Gq protein may induce additional conformational changes and/or prolong the dwell time of receptors in the active conformation. The dwell time in the active conformation can be evaluated by monitoring the transition from the agonist-bound active to agonist-free quiescent conformation as the FRET recovery upon the wash out of the agonists (Tateyama and Kubo 2013). We thus examined the effects of the Gq binding on the transition between the active and quiescent conformation, by monitoring the kinetics of FRET changes by using photomultiplier tubes. Coexpression of the G_*α*q_G_β1_G_γ2_ subunits significantly decelerated that of the M_3_R construct. The recovery time constant (τ_rec_) in the absence and presence of the Gq subunits was 408 ± 20 msec (*n* = 6) and 601 ± 57 msec (*n* = 9), respectively. On the other hand, the speeds of the agonist-induced FRET decreases were not altered by coexpression of the G_*α*q_G_β1_G_γ2_ subunits (Fig. 7). These results that Gq subunits-induced deceleration of the FRET recovery without affecting the speed of the FRET decrease accounted for the Gq-induced leftward shift of the concentration–FRET response curve. In addition, the deceleration of the FRET recovery induced by the Gq subunits suggested that the Gq binding stabilizes the active conformation of the M_3_R. In the case of the P2Y_1_R-i3-YFP/tail-CFP constructs, coexpression of the Gq subunits slightly but not significantly altered recovery kinetics; τ_rec_ was 1284 ± 177 msec (Gq-, *n* = 8) and 1570 ± 159 msec (Gq+, *n* = 9). The Gq binding was suggested not to sufficiently stabilize the active conformation of the P2Y_1_R.

The kinetic analysis revealed that the activation of the P2Y_1_R construct was slower than that of the M_3_R construct; τ_act_ was 852 ± 120 msec (Gq-, *n* = 8) for the P2Y_1_R-i3-YFP-long2/tail-CFP and 256 ± 31 msec (Gq-, *n* = 6) for the M_3_R-i3-YFP-long2/tail-CFP. It is possible that the slow activation of the P2Y_1_R construct is due to its intrinsic property or due to the property of the ADPβS. We thus monitored the FRET changes induced by the application of the full agonist (2MeSADP, 10 μmol/L). The activation kinetics by the 2MeSADP was not different from that by the ADPβS (τ_act_ was 696 ± 108 msec, Gq-, *n* = 5, Table3). These results suggested that the slow activation of P2Y1R construct is the intrinsic property of the construct.

## Discussion

### Evaluation of the functional Gq coupling of the i3-YFP receptors

In this study, the functional Gq coupling of the fluorescent receptor constructs was evaluated by measuring the decreases in the *I*_CFP-PH_ under the TIRF illumination (Fig. 1), instead of monitoring increases in the intracellular calcium concentration ([Ca^2+^]_i_). This was to avoid the problem due to the low-level presence of the endogenous P2YRs, as mentioned in the results. Activation of the endogenous P2YRs markedly increased the [Ca^2+^]_i_ but failed to decrease the *I*_CFP-PH_ (data not shown), indicating that the sensitivity to the Gq-induced PIP_2_ breakdown was different between these two monitoring methods; slight breakdown of the PIP_2_ sufficiently causes remarkable increases in the [Ca^2+^]_i_ but not detectable decreases in the *I*_CFP-PH_. This was also true for the NK_1_R-i3-YFP-long2 constructs, which slightly decreased the *I*_CFP-PH_ (Fig. 1) and clearly increased the [Ca^2+^]_i_ (data not shown). In addition, the decreases in the *I*_CFP-PH_ sustained after the wash out of the agonist of SP, which was consistent with the quite slow unbinding of the SP from the NK_1_R (Aharony et al. 1991; Roosterman et al. 2007). Therefore, monitoring the decreases in the *I*_CFP-PH_ was judged to be appropriate to quantitatively and qualitatively evaluate the functional Gq coupling of the fluorescent receptors.

### Effects of the junctional linkers on the function of the i3-YFP receptors

We examined two junctional linkers to connect YFP with the i3 of the receptors, and found that the junctional linker derived from the M_1_R (long2) served as spacers more effectively than that of the conventional flexible one (long1) both for the P2Y_1_R- and M_3_R-i3-YFP (Fig. 1). Because their lengths were similar to each other (Fig. 1B), the flexibility given by the long1 linker might confer unfavorable effects on the Gq binding of the i3-YFP receptors. When the long1 linkers were inserted instead of the long2 in the M_3_R FRET constructs (M_3_R-i3-YFP-long1/tail-CFP), the agonist-induced FRET decrease and its recovery upon the agonist washout were markedly slower than those observed in the short and long2 constructs (data not shown). From these results, the flexible linkers were thought to loosen the structural interaction between the TM5&6 and YFP. In contrast, the long2 linker was suggested to be functionally rigid enough to effectively couple the rearrangements of the TM with the i3-YFP. The long2 linker fully restored the intact function of the P2Y_1_R and the M_3_R but not of the NK_1_R, indicating that this linker cannot be used to all Gq-coupled receptors for the fusion of the FP. The linkers which do not disrupt the intact function of all Gq-coupled receptors, including the NK_1_R, are still awaited.

### Effects of the Gq binding on the activated conformation of the M_3_R

Coexpression of Gq protein was shown to enhance the agonist-induced decreases in FRET of the M_3_R-i3-YFP-long2/tail-CFP (Figs. 3 and 6) similarly with the M_1_R construct (Tateyama and Kubo 2013). As the maximal amplitudes of the ΔnF/*I*_CFP_ were significantly increased by the Gq subunits at the saturating concentration of the agonist (Fig. 6), the agonist-bound activated conformation of the M_3_R was suggested to be different from the conformation of the agonist- and Gq-bound M_3_R. As a recent spectroscopic study of the β-adrenergic receptors suggested that the agonist binding does not stabilize the fully activated conformation of the receptor (Nygaard et al. 2013), the binding of Gq protein was thought to stabilize the fully activated conformation of the M_3_R.

Coexpression of Gq protein also shifted the concentration–response curve toward left and decelerated the recovery of the decreased FRET of the M_3_R upon the washout of the agonist (Figs. 6 and 7). These effects on the concentration–response curve and the recovery speed were similar to those observed in the recent FRET studies of the M_3_R (Hoffmann et al. 2012) using a constitutively active mutant (Dowling et al. 2006). These results were consistent with the notion that the M_3_R stays in the activated state by the constitutive active mutation and by the binding of Gq protein, and that the binding of Gq protein stabilizes the active conformation of the M_3_R.

### Difference in the effects of the Gq binding on the activated conformation of the muscarinic receptors and P2Y_1_R

In this study, it was revealed that the coexpression of the Gq subunits did not significantly enhance the FRET decrease of the P2Y_1_R-i3-YFP-long2/tail-CFP and not significantly decelerate the FRET recovery (Figs. 3, 6, and 7). As the construct was functionally intact, those results were not due to the lack of the Gq binding. However, the maximal amplitude of the decreases in the *I*_CFP-PH_ induced by the activation of P2Y_1_R construct was almost half of those by activation of the M_3_R one (Fig. 1). A similar difference was observed when wild-type receptors were activated, suggesting that the binding of Gq protein with the P2Y_1_R is inefficient when compared to that with the M_3_R. The inefficient binding suggested that the interaction between the P2Y_1_R and the Gq subunits was not strong and fraction of the A/P2Y_1_R*/Gq complex is small. The unstable interaction between the P2Y_1_R and Gq protein may account in partly for the slight effects of the Gq binding on the changes in FRET. In addition, the lack of the Gq-induced enhancement of the ΔnF/*I*_CFP_ at the saturating concentration of the agonist suggested that the agonist-bound activated conformation of the P2Y_1_R was similar to the conformation of the agonist- and Gq-bound receptor. It could be further speculated that the agonist binding may induce the fully activated conformation of the P2Y_1_R without the binding of G protein, as suggested in metarhodopsin II (Choe et al. 2011).

In conclusion, binding of Gq protein significantly stabilizes the active conformation of the M_3_R but not of the P2Y_1_R. These results suggest that effects of the binding of G protein on the active conformation of GPCR may differ depending on the type of GPCR.
